# Routine 36‐week scan: diagnosis and outcome of abnormal fetal presentation

**DOI:** 10.1002/uog.29139

**Published:** 2024-12-02

**Authors:** M. Fitiri, D. Papavasileiou, V. Mesaric, A. Syngelaki, R. Akolekar, K. H. Nicolaides

**Affiliations:** ^1^ Fetal Medicine Research Institute King's College Hospital London UK; ^2^ Fetal Medicine Unit Medway Maritime Hospital Gillingham UK; ^3^ Institute of Medical Sciences Canterbury Christ Church University Chatham UK

**Keywords:** breech presentation, cephalic presentation, external cephalic version, non‐cephalic presentation, oblique presentation, prenatal diagnosis, pyramid of pregnancy care, third‐trimester screening, transverse presentation, ultrasound examination

## Abstract

**Objectives:**

First, to report the incidence of non‐cephalic presentation at a routine 36‐week ultrasound scan, the uptake and success of external cephalic version (ECV) and the incidence of spontaneous rotation from non‐cephalic to cephalic presentation. Second, to determine the maternal and pregnancy characteristics that provide a significant contribution to the prediction of non‐cephalic presentation at the 36‐week scan, successful ECV from non‐cephalic to cephalic presentation and spontaneous rotation from non‐cephalic to cephalic presentation.

**Methods:**

This was a retrospective analysis of prospectively collected data from 107 875 women with a singleton pregnancy who had undergone a routine ultrasound scan at 35 + 0 to 36 + 6 weeks' gestation. Patients with breech or transverse/oblique presentation were divided into two groups: those scheduled for elective Cesarean section for a fetal or maternal indication other than abnormal presentation, and those that would potentially require ECV. The latter group was reassessed after 1–2 weeks and, if the abnormal presentation persisted, the parents were offered ECV or elective Cesarean section at 38–40 weeks' gestation. Multivariable logistic regression analysis was carried out to determine which maternal and pregnancy characteristics provided a significant contribution in the prediction of non‐cephalic presentation at the 36‐week scan, successful ECV from non‐cephalic to cephalic presentation and spontaneous rotation from non‐cephalic to cephalic presentation.

**Results:**

At the 36‐week scan, fetal presentation was cephalic in 101 664 (94.2%) pregnancies and either breech, transverse or oblique in 6211 (5.8%). In 0.3% of cases with cephalic presentation at the 36‐week scan, there was subsequent spontaneous rotation to non‐cephalic presentation, and in half of these, the diagnosis was made during labor or at birth. ECV was attempted in 1584/6211 (25.5%) pregnancies with non‐cephalic presentation at the 36‐week scan and was successful in only 44.1% of cases. In the remaining 74.5% of cases, ECV was not attempted because of any of the following reasons: ECV was declined; Cesarean section was planned for a reason other than abnormal presentation; ECV was planned for the subsequent 1–2 weeks but, in the meantime, there was spontaneous rotation to cephalic presentation; or there was spontaneous onset of labor or rupture of membranes before planned ECV. In 5513/6211 (88.8%) pregnancies with non‐cephalic presentation at the 36‐week scan, ECV was not attempted or was unsuccessful, and in 37.7% of these, there was subsequent spontaneous rotation to cephalic presentation. Among the 6211 pregnancies with non‐cephalic presentation at the 36‐week scan, the presentation at birth was cephalic in 43.8%; in 74.8%, this was due to spontaneous rotation, and in 25.2%, it was due to successful ECV. Multivariable analysis demonstrated that the likelihood of non‐cephalic presentation at the 36‐week scan, that of successful ECV and that of spontaneous rotation from non‐cephalic to cephalic presentation was affected by several maternal and pregnancy characteristics, but the predictive performance for these events was poor, with the area under the receiver‐operating‐characteristics curve ranging from 0.608 to 0.717 and the detection rate at a 10% false‐positive rate ranging from 19.0% to 33.7%.

**Conclusions:**

Routine ultrasound examination at 35 + 0 to 36 + 6 weeks' gestation could improve pregnancy outcome by substantially reducing the risk of unexpected abnormal presentation in labor. However, an additional ultrasound scan for fetal presentation should be considered in all women when they present in labor. © 2024 The Author(s). *Ultrasound in Obstetrics & Gynecology* published by John Wiley & Sons Ltd on behalf of International Society of Ultrasound in Obstetrics and Gynecology.

## INTRODUCTION

Routine ultrasound examination at 35 + 0 to 36 + 6 weeks' gestation is useful for the prediction of small and large neonates^1^, ^2^, ^3^, ^4^, ^5^, diagnosis of previously undetected fetal abnormalities^6^, ^7^, assessment of the risk for pre‐eclampsia and reduction of such risk by timed birth^8^, ^9^, ^10^, prediction of adverse perinatal outcome^11^, ^12^, and diagnosis and management of non‐cephalic presentation^13^, ^14^, ^15^.

Undiagnosed non‐cephalic presentation in labor is associated with an increased risk of adverse outcome for both the mother and the baby, and a major randomized controlled trial (RCT) reported that breech vaginal delivery is associated with higher perinatal mortality and morbidity compared with breech elective Cesarean section^16^. After publication of this study, there was a shift toward performing elective Cesarean section when breech presentation was detected at term, with the rate increasing from about 50% to more than 90%^17^, ^18^. However, this rise in Cesarean section was associated with an increased risk of short‐ and long‐term maternal and fetal complications^19^, ^20^. Consequently, the Royal College of Obstetricians and Gynaecologists and the American College of Obstetricians and Gynecologists recommend that external cephalic version (ECV) should be offered to all eligible woman diagnosed with breech presentation at term in order to reduce non‐cephalic presentation at delivery and the rate of Cesarean section^21^, ^22^, ^23^, ^24^. However, a high proportion of breech presentations at term are not detected by routine abdominal palpation and, therefore, the rate of undiagnosed breech presentation in labor remains relatively high^14^, ^25^, ^26^, ^27^.

In a previous study, we reported on fetal presentation and pregnancy management in 45 847 singleton pregnancies that had undergone a routine 36‐week scan^13^. Fetal presentation was non‐cephalic in 5.3% of cases. In about 20% of those cases, a Cesarean section was planned for an indication other than abnormal presentation. Among those eligible for ECV, only 49% of women accepted the procedure, and it was successful in 39% of cases.

The objectives of this study, which is considerably larger than our previous one^13^, were: first, to report the incidence of non‐cephalic presentation at a routine 36‐week scan, the uptake and success of ECV and the incidence of spontaneous rotation from non‐cephalic to cephalic presentation; and second, to determine the maternal and pregnancy characteristics that provide a significant contribution to the prediction of non‐cephalic presentation at the 36‐week scan, successful ECV from non‐cephalic to cephalic presentation and spontaneous rotation from non‐cephalic to cephalic presentation.

## METHODS

### Study population and design

This was a retrospective analysis of prospectively collected data from 107 875 women with a singleton pregnancy who had undergone a routine ultrasound examination at 35 + 0 to 36 + 6 weeks' gestation at King's College Hospital, London, UK, or Medway Maritime Hospital, Gillingham, UK, between March 2014 and November 2023. During this visit, we recorded maternal demographic characteristics and medical history and carried out an ultrasound examination, which included assessment of fetal anatomy, fetal biometry for calculation of estimated fetal weight (EFW) using the formula of Hadlock *et al*.^28^, determination of fetal presentation (cephalic, breech or transverse/oblique) and placental position, measurement of the deepest vertical pocket of amniotic fluid and Doppler examination of the uterine, umbilical and fetal middle cerebral arteries. Gestational age was determined by the measurement of fetal crown–rump length at 11–14 weeks or fetal head circumference at 19–24 weeks^29^, ^30^. The ultrasound examinations were carried out by examiners who had obtained the Fetal Medicine Foundation (FMF) certificate of competence in ultrasound examination for fetal abnormalities. The inclusion criteria for the study were singleton pregnancy delivering a non‐malformed liveborn or stillborn neonate. We excluded pregnancies with aneuploidy and those with major fetal abnormality. As this study was a retrospective analysis of data derived from routine clinical examinations, ethics committee approval was not required.

Patients with breech or transverse/oblique presentation were divided into two groups: those scheduled for elective Cesarean section for a fetal or maternal indication other than abnormal presentation, and those that would potentially require ECV. The latter group was reassessed after 1–2 weeks and, if the abnormal presentation persisted, the parents were offered ECV or elective Cesarean section at 38–40 weeks' gestation. ECV was carried out by obstetricians or trained midwives at 37–38 weeks' gestation under ultrasound guidance and after the administration of terbutaline (0.25 mg subcutaneously).

Data on pregnancy outcome were collected from the hospital maternity records and included gestational age at delivery, method of onset of labor and delivery, fetal presentation at birth and birth weight. EFW percentile was derived from the FMF fetal and neonatal population weight charts^31^.

### Statistical analysis

Data were expressed as median (interquartile range) for continuous variables and *n* (%) for categorical variables. The Mann–Whitney *U*‐test and the chi‐square test or Fisher's exact test were used for comparing outcome groups for continuous and categorical data, respectively. Significance was assumed at 5%.

Univariable and multivariable logistic regression analyses were carried out to determine which maternal and pregnancy characteristics provided a significant contribution to the prediction of non‐cephalic presentation at the 36‐week scan, successful ECV from non‐cephalic to cephalic presentation and spontaneous rotation from non‐cephalic to cephalic presentation. Prior to the regression analysis, maternal age, weight and height were centered by subtracting the arithmetic mean from each value. Multiple categorical variables were dummy coded as binary variables to estimate the independent effect of each category. Predicted probabilities from logistic regression analysis were used to construct receiver‐operating‐characteristics (ROC) curves to assess the performance of screening for each outcome. SPSS software version 24.0 (IBM Corp., Armonk, NY, USA) was used for statistical analysis.

## RESULTS

### Patient characteristics

The study population of 107 875 singleton pregnancies included 101 664 (94.2%) with cephalic presentation at the 35 + 0 to 36 + 6‐week ultrasound examination, 5243 (4.9%) with breech presentation and 968 (0.9%) with transverse or oblique presentation. Characteristics of the study population are summarized in Table 1. In the group with non‐cephalic presentation, compared to those with cephalic presentation, median maternal age, maternal weight and EFW percentile were higher, and there was a higher proportion of white women, nulliparas, women with a previa, fundal or lateral placental location and those with oligo‐ or polyhydramnios.

**Table 1:** Maternal and pregnancy characteristics of study population (*n* = 107 875), according to fetal presentation at routine ultrasound scan at 36 weeks' gestation

Variable	Cephalic presentation (*n* = 101 664)	Non‐cephalic presentation (*n* = 6211)	*P*
Transverse or oblique presentation	—	968 (15.6)	—
Maternal age (years)	32.0 (28.0–35.6)	33.4 (29.7–36.9)	< 0.001
Maternal weight (kg)	79.1 (70.9–90.0)	80.0 (71.2–92.0)	0.049
Maternal height (cm)	165 (161–167)	165 (161–170)	0.267
Race			
White	76 045 (74.8)	4800 (77.3)	< 0.001
Black	14 803 (14.6)	851 (13.7)	0.062
South Asian	5649 (5.6)	308 (5.0)	0.045
East Asian	1986 (2.0)	123 (2.0)	0.882
Mixed	3181 (3.1)	129 (2.1)	< 0.001
Parity			
Nulliparous	46 536 (45.8)	3005 (48.4)	< 0.001
Parous	55 128 (54.2)	3206 (51.6)	< 0.001
GA at ultrasound (weeks)	36.0 (35.6–36.3)	35.9 (35.6–36.3)	0.280
EFW percentile	54.1 (30.1–75.8)	57.8 (31.7–80.5)	0.008
Placental position			
Anterior	38 659 (38.0)	2194 (35.3)	< 0.001
Posterior	32 647 (32.1)	1745 (28.1)	< 0.001
Fundal/lateral	30 029 (29.5)	2160 (34.8)	< 0.001
Previa	329 (0.3)	112 (1.8)	< 0.001
Amniotic fluid DVP			
< 2 cm	97 (0.1)	24 (0.4)	< 0.001
2–7 cm	100 000 (98.4)	5994 (96.5)	< 0.001
≥ 8 cm	1567 (1.5)	193 (3.1)	< 0.001

Data are given as median (interquartile range) or *n* (%).

DVP, deepest vertical pocket; EFW, estimated fetal weight; GA, gestational age.

### Findings at 36‐week scan and subsequent pregnancy management

#### Cephalic presentation

Among the 101 664 pregnancies with cephalic presentation at the 36‐week scan, 99.7% remained cephalic at birth (Table 2). In the remaining 0.3% of cases, there was subsequent spontaneous rotation to non‐cephalic presentation and, in 50.5% of these, the diagnosis was made during labor or at birth. Among the 49.5% of pregnancies in which the diagnosis of non‐cephalic presentation was made before labor, ECV was declined in 70.8% cases. In 9.3% of cases, ECV was attempted but failed; in 13.0%, ECV was not attempted because Cesarean section was planned for a reason other than abnormal presentation; and in 6.8%, ECV was planned but, in the meantime, there was spontaneous rotation to cephalic presentation.

**Table 2:** Fetal presentation at 36 weeks' gestation and subsequent management (*n* = 107 875)

Variable	Value
*Cephalic presentation at 36‐week scan* (n *= 101 664*)	
Remained cephalic at birth	101 339/101 664 (99.7)
Spontaneous rotation to non‐cephalic	325/101 664 (0.3)
Diagnosis during labor or at birth	164/325 (50.5)
Diagnosis before labor	161/325 (49.5)
ECV attempted	15/161 (9.3)
Success	0/15 (0)
Failure	15/15 (100)
ECV planned but spontaneous rotation to cephalic	11/161 (6.8)
Remained cephalic	6/11 (54.5)
Spontaneous rotation to non‐cephalic	5/11 (45.5)
ECV declined	114/161 (70.8)
Remained non‐cephalic	113/114 (99.1)
Spontaneous rotation to cephalic	1/114 (0.9)
No ECV because CS planned for other indication	21/161 (13.0)
Remained non‐cephalic	21/21 (100)
*Non‐cephalic presentation at 36‐week scan* (n *= 6211*)	
ECV attempted	1584/6211 (25.5)
Success	698/1584 (44.1)
Remained cephalic	679/698 (97.3)
Spontaneous rotation to non‐cephalic	19/698 (2.7)
Repeat ECV, successful rotation to cephalic	7/19 (36.8)
Remained non‐cephalic	12/19 (63.2)
Diagnosis during labor or at birth	2/12 (16.7)
Failure	886/1584 (55.9)
Remained non‐cephalic	866/886 (97.7)
Spontaneous rotation to cephalic	20/886 (2.3)
ECV planned but spontaneous rotation to cephalic	1350/6211 (21.7)
Remained cephalic	1316/1350 (97.5)
Subsequent spontaneous rotation to non‐cephalic	34/1350 (2.5)
Diagnosis during labor or at birth	15/34 (44.1)
ECV planned but went into labor or SROM before ECV	124/6211 (2.0)
Remained non‐cephalic	122/124 (98.4)
Spontaneous rotation to cephalic	2/124 (1.6)
ECV declined	2238/6211 (36.0)
Remained non‐cephalic	1703/2238 (76.1)
Spontaneous rotation to cephalic	535/2238 (23.9)
Remained cephalic	526/535 (98.3)
Subsequent spontaneous rotation to non‐cephalic	9/535 (1.7)
Diagnosis during labor or at birth	6/9 (66.7)
No ECV because CS planned for other indication	915/6211 (14.7)
Spontaneous rotation to cephalic	172/915 (18.8)
Remained non‐cephalic	743/915 (81.2)
Diagnosis during labor or at birth	2/743 (0.3)

Data are given as *n/N* (%).

CS, Cesarean section; ECV, external cephalic version; SROM, spontaneous rupture of membranes.

#### Non‐cephalic presentation

Among the 6211 pregnancies with non‐cephalic presentation at the 36‐week scan, ECV was attempted in only 25.5% of cases, and was successful in only 44.1% of these (Table 2). In the remaining 74.5% of cases, ECV was not attempted because of any of the following reasons: ECV was declined; Cesarean section was planned for a reason other than abnormal presentation; ECV was planned for the subsequent 1–2 weeks but, in the meantime, there was spontaneous rotation to cephalic presentation; or there was spontaneous onset of labor or rupture of membranes before planned ECV.

In the 6211 pregnancies with non‐cephalic presentation at the 36‐week scan, the presentation at birth was cephalic in 2722 (43.8%). In 2036 (74.8%), this was due to spontaneous rotation, and in 686 (25.2%), it was due to successful ECV.

### Prediction of non‐cephalic presentation at 36‐week scan

In the total population of 107 875 singleton pregnancies, logistic regression analysis was carried out to determine which maternal and pregnancy characteristics provided a significant contribution to the prediction of non‐cephalic presentation at the 36‐week scan (Table 3). The following variables were examined: maternal age, weight, height, race and parity (nulliparous or parous), gestational age at ultrasound, EFW percentile, placental position (previa or non‐previa) and amniotic fluid deepest vertical pocket.

**Table 3:** Fitted regression model with maternal and pregnancy characteristics for prediction of non‐cephalic presentation at 36‐week scan

	Univariable		Multivariable	
Characteristic	OR (95% CI)	*P*	aOR (95% CI)	*P*
Maternal age – 32 (in years)	1.053 (1.048–1.058)	< 0.001	1.056 (1.051–1.062)	< 0.001
Maternal weight – 82 (in kg)	1.005 (1.003–1.006)	< 0.001	1.005 (1.004–1.007)	< 0.001
Maternal height – 165 (in cm)	1.001 (0.997–1.005)	0.558	0.991 (0.987–0.995)	< 0.001
Race				
White	Reference		Reference	
Black	0.911 (0.845–0.982)	0.015	0.897 (0.831–0.969)	0.006
South Asian	0.864 (0.767–0.972)	0.015	0.816 (0.723–0.921)	0.001
East Asian	0.981 (0.816–1.180)	0.840	0.894 (0.741–1.079)	0.242
Mixed	0.642 (0.537–0.768)	< 0.001	0.651 (0.545–0.779)	< 0.001
Parity				
Nulliparous	Reference		Reference	
Parous	0.901 (0.856–0.948)	< 0.001	0.781 (0.741–0.824)	< 0.001
GA at ultrasound (in weeks)	0.864 (0.825–0.904)	< 0.001	0.855 (0.817–0.895)	< 0.001
Estimated fetal weight				
10–90^th^ percentile	Reference		Reference	
< 10^th^ percentile	1.159 (1.057–1.270)	0.002	1.274 (1.159–1.400)	< 0.001
> 90^th^ percentile	1.533 (1.421–1.655)	< 0.001	1.352 (1.248–1.446)	< 0.001
Placental position				
Non‐previa	Reference		Reference	
Previa	5.565 (4.558–7.020)	< 0.001	4.889 (3.928–6.086)	< 0.001
Amniotic fluid DVP				
2–7 cm	Reference		Reference	
< 2 cm	4.128 (2.638–6.458)	< 0.001	4.058 (2.575–6.395)	< 0.001
≥ 8 cm	2.055 (1.765–2.392)	< 0.001	1.698 (1.450–1.987)	< 0.001

aOR, adjusted odds ratio; DVP, deepest vertical pocket; GA, gestational age; OR, odds ratio.

Multivariable analysis demonstrated that the likelihood of non‐cephalic presentation increased with increasing maternal age and weight, decreasing maternal height and earlier gestational age at ultrasound. Non‐cephalic presentation was more likely in the presence of placenta previa, oligohydramnios, polyhydramnios, EFW < 10^th^ percentile and EFW > 90^th^ percentile, and was less likely in parous women compared with nulliparous women, and in women of black, South Asian or mixed racial origin compared with white women. The predictive performance was poor, with an *R* ^2^ of 0.026, an area under the ROC curve (AUC) of 0.608 (95% CI, 0.601–0.616) and a detection rate of 19.0% (95% CI, 18.1–20.0%) at a false‐positive rate (FPR) of 10%.

### Successful ECV from non‐cephalic to cephalic presentation

In the 1584 cases with non‐cephalic presentation at the 36‐week scan in which ECV was attempted, logistic regression analysis was carried out to determine which maternal and pregnancy characteristics provided a significant contribution to the prediction of successful ECV (Table 4). The following variables were examined: maternal age, weight, height, race and parity (nulliparous or parous), type of non‐cephalic presentation (breech or transverse/oblique), EFW percentile, placental position (anterior, posterior or lateral/fundal) and amniotic fluid deepest vertical pocket.

**Table 4:** Fitted regression model with maternal and pregnancy characteristics for prediction of successful external cephalic version from non‐cephalic to cephalic presentation

	Univariable		Multivariable	
Characteristic	OR (95% CI)	*P*	aOR (95% CI)	*P*
Maternal age – 32 (in years)	1.022 (1.002–1.042)	0.032	—	—
Maternal weight – 82 (in kg)	0.997 (0.991–1.003)	0.329	0.988 (0.982–0.995)	0.001
Maternal height – 165 (in cm)	0.993 (0.978–1.009)	0.390	—	—
Race				
White	Reference			
Black	1.319 (0.966–1.802)	0.082	—	—
South Asian	0.955 (0.588–1.549)	0.851	—	—
East Asian	1.180 (0.628–2.217)	0.606	—	—
Mixed	0.771 (0.385–1.544)	0.463	—	—
Parity				
Nulliparous	Reference		Reference	
Parous	3.085 (2.502–3.804)	< 0.001	3.062 (2.461–3.810)	< 0.001
Non‐cephalic presentation				
Breech	Reference		Reference	
Transverse or oblique	2.385 (1.404–4.051)	0.001	1.813 (1.034–3.179)	0.038
Estimated fetal weight				
10–90^th^ percentile	Reference		Reference	
< 10^th^ percentile	0.528 (0.329–0.847)	0.008	0.451 (0.272–0.750)	0.002
> 90^th^ percentile	0.921 (0.651–1.303)	0.642	—	—
Placental position				
Posterior	Reference		Reference	
Anterior	0.844 (0.655–1.086)	0.188	0.750 (0.575–0.978)	0.034
Lateral or fundal	1.997 (1.558–2.561)	< 0.001	1.773 (1.366–2.302)	< 0.001
Amniotic fluid DVP				
2–7 cm	Reference			
< 2 cm	—*	—*	—	—
≥ 8 cm	1.013 (0.398–2.581)	0.978	—	—

* No patients had oligohydramnios in this analysis.

aOR, adjusted odds ratio; DVP, deepest vertical pocket; OR, odds ratio.

Multivariable analysis demonstrated that the likelihood of successful ECV decreased with increasing maternal weight. Successful ECV was more likely in parous women compared with nulliparous women, in those with transverse or oblique fetal presentation compared to those with breech presentation, and in the presence of a lateral or fundal placenta compared with a posterior placenta. The likelihood of successful ECV was lower if the EFW was < 10^th^ percentile or if the placental position was anterior. The predictive performance was poor, with an *R* ^2^ of 0.145, an AUC of 0.701 (95% CI, 0.676–0.727) and a detection rate of 28.8% (95% CI, 25.5–32.3%) at a FPR of 10%.

### Spontaneous rotation from non‐cephalic to cephalic presentation

In 5513/6211 (88.8%) pregnancies with non‐cephalic presentation at the 36‐week scan, ECV was not attempted or was unsuccessful (Table 2). In 2079/5513 (37.7%) pregnancies, there was spontaneous rotation to cephalic presentation. Logistic regression analysis was performed to determine which maternal and pregnancy characteristics provided a significant contribution to the prediction of spontaneous rotation from non‐cephalic to cephalic presentation (Table 5). The following variables were examined: maternal age, weight, height, race and parity (nulliparous or parous), type of non‐cephalic presentation (breech or transverse/oblique), EFW percentile, placental position (previa or non‐previa) and amniotic fluid deepest vertical pocket.

**Table 5:** Fitted regression model with maternal and pregnancy characteristics for prediction of spontaneous rotation from non‐cephalic to cephalic presentation

	Univariable		Multivariable	
Characteristic	OR (95% CI)	*P*	aOR (95% CI)	*P*
Maternal age – 32 (in years)	1.026 (1.016–1.036)	< 0.001	—	—
Maternal weight – 82 (in kg)	1.007 (1.004–1.010)	< 0.001	—	—
Maternal height – 165 (in cm)	1.005 (0.996–1.013)	0.272	1.018 (1.009–1.027)	< 0.001
Race				
White	Reference		Reference	
Black	1.865 (1.596–2.179)	< 0.001	1.255 (1.059–1.488)	0.009
South Asian	1.222 (0.953–1.567)	0.115	—	—
East Asian	0.842 (0.553–1.283)	0.424	—	—
Mixed	1.290 (0.886–1.880)	0.184	—	—
Parity				
Nulliparous	Reference		Reference	
Parous	3.367 (2.997–3.782)	< 0.001	2.826 (2.501–3.194)	< 0.001
Non‐cephalic presentation				
Breech	Reference		Reference	
Transverse or oblique	4.431 (3.814–5.148)	< 0.001	3.665 (3.110–4.318)	< 0.001
Estimated fetal weight				
10–90^th^ percentile	Reference		Reference	
< 10^th^ percentile	0.590 (0.480–0.726)	< 0.001	0.752 (0.604–0.935)	0.011
> 90^th^ percentile	1.102 (0.942–1.289)	0.223	0.757 (0.635–0.903)	0.002
Placental position				
Non‐previa	Reference		Reference	
Previa	0.709 (0.471–1.069)	0.100	0.329 (0.211–0.515)	< 0.001
Amniotic fluid DVP				
2–7 cm	Reference		Reference	
< 2 cm	0.160 (0.037–0.679)	0.013	0.168 (0.038–0.746)	0.019
≥ 8 cm	2.462 (1.828–3.316)	< 0.001	1.870 (1.347–2.595)	< 0.001

aOR, adjusted odds ratio; DVP, deepest vertical pocket; OR, odds ratio.

Multivariable analysis demonstrated that the likelihood of spontaneous rotation from non‐cephalic to cephalic presentation increased with increasing maternal height, and was higher in black women compared with white women, in parous women compared with nulliparous women, in those with transverse or oblique fetal presentation compared to those with breech presentation, and in the presence of polyhydramnios. The likelihood of spontaneous rotation from non‐cephalic to cephalic presentation was lower if the placenta was previa, if there was oligohydramnios and if the EFW was < 10^th^ percentile or > 90^th^ percentile. The predictive performance was poor, with an *R* ^2^ of 0.181, an AUC of 0.717 (95% CI, 0.703–0.731) and a detection rate of 33.7% (95% CI, 31.7–35.8%) at a FPR of 10%.

## DISCUSSION

### Main findings

There are six main findings of our study in a heterogeneous population of women with a singleton pregnancy undergoing a routine ultrasound examination at 35 + 0 to 36 + 6 weeks' gestation. First, fetal presentation was cephalic in about 95% of pregnancies, and either breech, transverse or oblique in 5%. Second, in 0.3% of the cases with cephalic presentation at the 36‐week scan, there was subsequent spontaneous rotation to non‐cephalic presentation and, in half of these, the diagnosis was made during labor or at birth. Third, ECV was attempted in 26% of cases with non‐cephalic presentation at the 36‐week scan, but was successful in only around 45% of these. In the remaining 74% of cases, ECV was not attempted because of any of the following reasons: ECV was declined; Cesarean section was planned for a reason other than abnormal presentation; ECV was planned for the subsequent 1–2 weeks but, in the meantime, there was spontaneous rotation to cephalic presentation; or there was spontaneous onset of labor or rupture of membranes before planned ECV. Fourth, in around 90% of cases with non‐cephalic presentation at the 36‐week scan, ECV was not attempted or was unsuccessful, and in 38% of these, there was subsequent spontaneous rotation to cephalic presentation. Fifth, among pregnancies with non‐cephalic presentation at the 36‐week scan, the presentation at birth was cephalic in 44% of cases; in 75%, this was due to spontaneous rotation, and in 25%, due to successful ECV. Sixth, multivariable analysis demonstrated that the likelihood of non‐cephalic presentation at the 36‐week scan, that of successful ECV and that of spontaneous rotation from non‐cephalic to cephalic presentation was affected by several maternal and pregnancy characteristics, but the predictive performance for these events was poor, with AUCs ranging from 0.608 to 0.717 and detection rates at a 10% FPR ranging from 19.0% to 33.7%.

### Comparison with literature

#### Incidence of non‐cephalic presentation at 36 weeks' gestation

Wastlund *et al*. reported that, among 3879 women with a singleton pregnancy who underwent routine ultrasound examination at 36 weeks' gestation, the incidence of breech presentation was 4.6%^14^. In the group with breech presentation, compared to those with cephalic presentation, maternal age was higher, but there was no significant difference in body mass index or birth‐weight percentile^14^. In our considerably larger study, which included both nulliparous and parous women, the incidence of non‐cephalic presentation was similar but, in addition to increased maternal age, there were many other significant contributors to abnormal presentation at 36 weeks, including increased maternal weight and decreased maternal height.

A Finnish study of 737 788 women with a singleton pregnancy delivering at 24–42 weeks' gestation reported that the incidence of breech presentation at delivery decreased from 23.5% at 24–27 weeks to 2.5% in term pregnancies^32^. Risk factors for breech presentation in term pregnancies included advanced maternal age, nulliparity, placenta previa, preterm prelabor rupture of membranes, oligohydramnios, congenital anomaly and birth weight < 10^th^ percentile.

#### Accuracy of diagnosis of non‐cephalic presentation by abdominal palpation

In our study, we did not record the findings from routine clinical examination before the ultrasound scan. Studies undertaken as part of a research protocol in which clinical examination was followed by an ultrasound scan reported that abdominal palpation correctly identified non‐cephalic presentation in 57–70% of cases^25^, ^26^; however, the design of such studies is likely to have introduced positive bias in favor of clinical examination. A more realistic estimate of the accuracy of routine clinical examination in the detection of non‐cephalic presentation at 36 weeks' gestation is 44%, as reported by Wastlund *et al*.^14^. Another study, by Melo *et al*., reported that ultrasound examination confirmed non‐cephalic presentation in only 41% of 7775 pregnancies suspected of breech presentation during routine antenatal care^27^.

#### Acceptability and success of ECV

Among the 6211 cases with non‐cephalic presentation at the 36‐week scan included in the present study, ECV was not considered in 915 cases because Cesarean section was planned for another indication (Table 2). Of the remaining 5296 patients who were eligible for ECV, 2238 (42.3%) declined the procedure; among the 3058 (57.7%) who agreed, ECV was not attempted in 1474 (48.2%) cases because of spontaneous rotation to cephalic presentation (*n* = 1350) or spontaneous onset of labor or rupture of membranes (*n* = 124) before planned ECV. Consequently, ECV was attempted in only 1584/3058 (51.8%) women who agreed to undergo this procedure, and it was successful in only 698/1584 (44.1%) cases. Of note, our patients with non‐cephalic presentation at the 36‐week scan were counseled and managed by their own obstetricians and midwives rather than in a dedicated clinic.

In the study of Wastlund *et al*., the uptake of ECV among eligible women was 65%, and this was successful in only 14% of cases^14^. In contrast, in the study of Melo *et al*., in which all women with breech presentation were managed in a specialist clinic, the uptake of ECV among eligible women was 90% and this was successful in 49% of cases^27^, which is not substantially different from the 44.1% success rate in our non‐specialist service.

In relation to the timing of ECV, a Cochrane review of three RCTs reported that the success rate is higher if ECV is carried out at 34–35 weeks' gestation, rather than at 37–38 weeks, but at the expense of a higher rate of preterm birth^33^.

We found that the factors contributing to successful ECV included low maternal weight, parity ≥ 1, transverse or oblique presentation rather than breech presentation and lateral or fundal placental position rather than anterior or posterior placenta. In a study from the USA of 149 671 women who underwent ECV, contributors to successful ECV were African American, American Indian or Native Alaskan race and low (< 25 years) or high (> 40 years) maternal age^34^. Similar to the present study, factors that contributed to failed ECV included nulliparity, high body mass index and low EFW^34^.

#### Spontaneous rotation from non‐cephalic to cephalic presentation

In our population of 6211 cases with non‐cephalic presentation at the 36‐week scan, ECV was successful in 698 (11.2%) cases and it was either not attempted or failed in 5513 (88.8%) cases. In 2079/5513 (37.7%) cases, there was subsequent spontaneous rotation to cephalic presentation. This is consistent with the findings of a registry study of 127 171 births, which reported that the frequency of breech presentation for births at 35–36 weeks' gestation was 4.9% and this declined to 3.6% for births at 37–38 weeks, 2.6% at 39–40 weeks and 1.7% at > 40 weeks^35^. In the study of Wastlund *et al*., there was spontaneous rotation from breech presentation at the 36‐week scan to cephalic presentation at delivery in 25/179 (14.0%) cases^14^.

In relation to the factors contributing to the prediction of spontaneous rotation from non‐cephalic to cephalic presentation, we found these to include high maternal height, parity ≥ 1, black rather than white racial origin, transverse or oblique presentation rather than breech presentation, and polyhydramnios.

#### Impact of 36‐week scan on incidence of non‐cephalic presentation at birth

In our study of 107 875 pregnancies, the incidence of non‐cephalic presentation at the 36‐week scan was 5.8% and that at the time of birth was 3.5%. This substantial decrease was mainly due to spontaneous rotation, rather than successful ECV. The rate of undiagnosed non‐cephalic presentation in labor was 0.2%.

Another study examined the impact of introducing a routine 36‐week scan on the incidence of breech presentation and undiagnosed breech presentation in women with a singleton pregnancy delivering in a single unit in Oxford, UK, between 37 + 0 and 42 + 6 weeks' gestation^15^. They compared the 2 years before (*n* = 14 444) and 2 years after (*n* = 13 381) the introduction of the routine 36‐week scan. Surprisingly, the incidence of breech presentation at birth did not change significantly (2.6% *vs* 2.7%), but the rate of undiagnosed breech presentation before labor was reduced from 22.3% to 4.7% (*P* < 0.001).

Hofmeyr *et al*. reviewed eight RCTs of ECV performed at or near term on a combined total of 1308 women with breech presentation and reported that ECV was associated with a significant reduction in non‐cephalic presentation at birth (risk ratio, 0.42 (95% CI, 0.29–0.61))^36^.

### Implications for clinical practice

Routine ultrasound examination at 35 + 0 to 36 + 6 weeks' gestation detects non‐cephalic presentation in about 5% of pregnancies. Such diagnosis could potentially improve pregnancy outcome by preventing unexpected abnormal presentation in labor. However, this study found that, in 0.3% of cases with cephalic presentation at the 36‐week scan, 2.5% of cases with non‐cephalic presentation at the 36‐week scan in which ECV was planned but there was spontaneous rotation to cephalic presentation, and 2.7% of those with non‐cephalic presentation at the 36‐week scan that had successful ECV, there was subsequent spontaneous rotation to non‐cephalic presentation. Consequently, the only strategy that would truly avoid unexpected non‐cephalic presentation in labor is to perform a routine ultrasound examination in all women on admission to the labor ward.

In our population of 107 875 women with a singleton pregnancy that had undergone a routine 36‐week scan, there were 189 (0.2%) cases with unexpected non‐cephalic presentation first detected during labor or at birth. This information can be used when planning future studies on the use of point‐of‐care ultrasound examination on admission to the labor ward in women that had undergone a routine 36‐week scan. Inevitably, the number of unexpected non‐cephalic presentations would be considerably higher in the absence of a routine 36‐week scan^15^, ^35^.

### Strengths and limitations

The strengths of our study are the examination of a large number of pregnancies undergoing a routine ultrasound examination at 35 + 0 to 36 + 6 weeks' gestation to determine fetal presentation, and the description of the subsequent management of pregnancies with abnormal presentation.

Limitations of the study include the lack of reporting of fetal presentation at routine clinical examination prior to the scan. There was no systematic recording at the time of the 36‐week scan of features that could contribute to abnormal presentation, including uterine malformation, myoma or presence of nuchal cord and the number of loops. Finally, there was no standardized protocol for the management of pregnancies with non‐cephalic presentation, which was left to the discretion of the attending obstetricians and midwives. Consequently, the uptake of ECV and success of the procedure are not generalizable.

### Conclusions

The performance of abdominal palpation during routine antenatal care in the diagnosis of non‐cephalic presentation at term is poor, resulting in a high proportion of such pregnancies being undiagnosed when they present in labor. Routine ultrasound examination at 35 + 0 to 36 + 6 weeks' gestation could improve pregnancy outcome by substantially reducing the risk of unexpected abnormal presentation in labor. However, an additional ultrasound scan for fetal presentation should be considered in all women when they present in labor.

## Data Availability

Research data are not shared.
